# Median nerve stimulation elevates ventricular fibrillation threshold *via* the cholinergic anti-inflammatory pathway in myocardial infarction canine model

**DOI:** 10.3389/fcvm.2022.904117

**Published:** 2022-12-01

**Authors:** Xuewen Wang, Yongsheng Qian, Yajun Yao, Youcheng Wang, Youjing Zhang, Shujuan Zhang, Qingyan Zhao

**Affiliations:** ^1^Department of Cardiology, Renmin Hospital of Wuhan University, Wuhan, China; ^2^Cardiovascular Research Institute of Wuhan University, Wuhan, China; ^3^Hubei Key Laboratory of Cardiology, Wuhan, China

**Keywords:** myocardial infarction, median nerve, sudden cardiac death, cholinergic anti-inflammatory pathway, arrhythmia

## Abstract

**Background:**

Median nerve stimulation (MNS) diminishes regional myocardial ischemia and ventricular arrhythmia; however, the underlying mechanism has not been elucidated.

**Methods:**

In this study, we randomly categorized 22 adult mongrel dogs into a control group, MNS group 1, and MNS group 2. After a 4-week experimental myocardial infarction (MI), ventricular electrophysiology was measured in the MNS group 1 before and after 30 min of MNS. The same measurements were performed in the MNS group 2 dogs *via* bilateral vagotomy. Venous blood and ventricular tissue were collected to detect molecular indicators related to inflammation and cholinergic pathways by enzyme-linked immunosorbent assay (ELISA), immunohistochemistry (IHC), and Western blot (WB).

**Results:**

No significant changes were reported in the ventricular effective refractory period (ERP) in the MNS group 1 and MNS group 2 dogs before and after MNS. The ventricular fibrillation threshold (VFT) in the MNS group 1 was significantly higher than that in the MNS group 2 (20.3 ± 3.7 V vs. 8.7 ± 2.9 V, *P* < 0.01). The levels of tumor necrosis factor-alpha (TNF-α), interleukin-6 (IL-6), and nuclear transcription factor-κB (NF-κB) were lower (*P* < 0.01), whereas the levels of Ach were higher in the peri-infarct zone tissues in the MNS group 1 dogs than those in the MNS group 2 dogs (*P* < 0.01).

**Conclusion:**

This study demonstrated that MNS increases VFT in a canine model with MI. The effects of MNS on VFT are potentially associated with the cholinergic anti-inflammatory pathway.

## Introduction

Sudden cardiac death (SCD)-related mortality is highest within the first-month post-acute myocardial infarction (MI) (1). In approximately 80% of cases, SCD is caused by sustained ventricular tachycardia (VT) and ventricular fibrillation (VF) secondary to acute MI (2). Previous studies reported the activity of the autonomic nervous system as an important factor for the initiation of ventricular arrhythmia (VA) during MI (3–5). Besides, increased innervation after myocardial injury may potentially result in increased sympathetic nerve density, consequently elevating the propensity for VA (6, 7). Decreased stellate ganglion nerve activity *via* spinal cord stimulation or renal denervation can pose beneficial effects on ventricular electrophysiology and VA (8–10). Stimulation of the vagus nerve, which acts antagonistically with the sympathetic nerve, can achieve anti-arrhythmic effects. Left-sided low-level vagus nerve stimulation (LLVNS) suppressed left stellate ganglion neural activity, especially in the morning, and decreased tyrosine-hydroxylase positive cells in the left stellate ganglion. Moreover, LLVNS prevented paroxysmal AF induced by rapid atrial pacing (11). In a subsequent study, the same investigators showed that chronic LLVNS damaged the stellate ganglia, resulting in reduced stellate ganglion nerve activity (12).

The median nerve is paired and arises from some of the spinal nerves of the cervical and thoracic regions. In our previous study, we found that stimulation of the median nerve causes a cardiac sympatho-vagal balance, decreasing stellate ganglion nerve activity, and enhancing cardiac vagal nerve activity (13). Median nerve stimulation (MNS) found to diminish regional myocardial ischemia and reduce ischemia-reperfusion-induced VA by decreasing cardiac metabolic demand (14, 15). This study aims to test the hypothesis that MNS suppresses VA vulnerability after MI and explore the mechanism of MNS effects on VA in a canine model with experimental MI.

## Materials and methods

### Animal model preparation

This experiment was approved by the animal studies subcommittee of our institutional review board and complied with the guidelines of the National Institutes of Health for the care and use of laboratory animals. A total of 22 adult mongrel dogs (weighing 15–18 kg) were used in the experiment. Before pentobarbital sodium premedication, we subjected the dogs to an intramuscular injection of 25 mg/kg ketamine sulfate. All dogs were premedicated with sodium pentobarbital (30 mg/kg, IV), intubated, and ventilated in a room atmosphere supplemented with oxygen from a respirator (MAO01746, Harvard Apparatus, Holliston, MA, USA). Continuous electrocardiogram (ECG) monitoring was performed.

**FIGURE 1:**
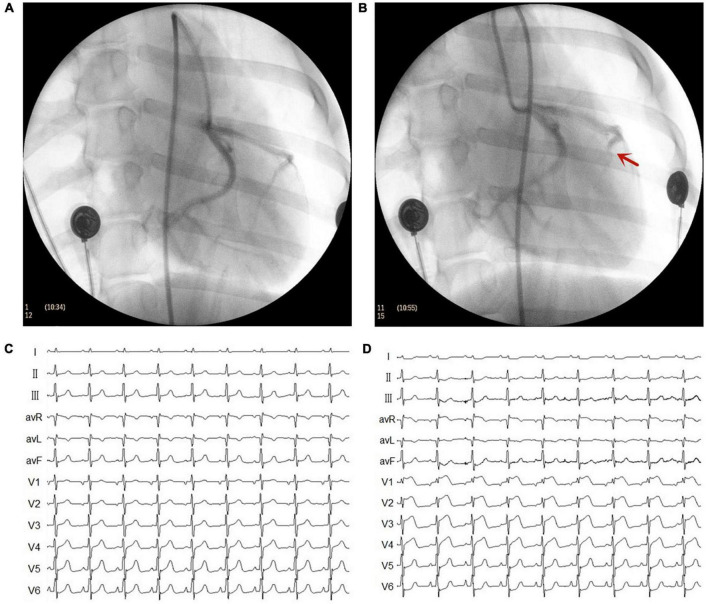
Representative images of X-ray and electrocardiogram (ECG) changes during the myocardial infarction (MI) model. **(A)** Left coronary artery arteriography before gelatin sponge injection. **(B)** Left coronary artery arteriography after gelatin sponge injection. Notably, the distal anterior descending artery had obvious stenosis. **(C)** ECG before gelatin sponge injection. **(D)** ECG after gelatin sponge injection, the chest lead ST segment was elevated.

**FIGURE 2:**
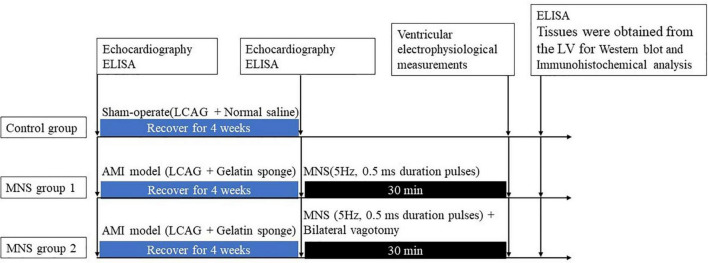
Median nerve stimulation protocol and time axis of procedures used in this study. LV, left ventricle; AMI, acute myocardial ischemia; LCAG, left coronary angiography; MNS, median nerve stimulation.

The MI model was established using catheter intervention. After administering stable anesthesia to all dogs, we injected 1000 U of heparin; then, we inserted hemostatic sheaths into their right femoral artery. Using X-ray fluoroscopy, a 5 F catheter was inserted into the left coronary artery to determine a coronary angiogram. Keeping the 5 F catheter positioned at the left anterior descending artery, a Radifocus SP catheter was delivered to the distal anterior descending artery through the 5 F catheter. A gelatin sponge with normal saline was injected into the distal anterior descending artery in 15 dogs. Notably, if the ST segment had no significant changes, the gelatin sponge with normal saline was reinjected. When the chest lead ST-segment was elevated, acute MI was achieved (Figure 1). The control group consisted of the other seven dogs injected with normal saline at the distal end of the anterior descending branch. Later, the animals were allowed to recover for 4 weeks. Figure 2 shows the time axis of procedures used in this study.

### Echocardiography

Transthoracic 2D and Doppler echocardiography were performed in all animals with Vivid 7 (GE, Boston, MA, USA) at baseline and after 4 weeks. Standard 2-D short- and long-parasternal views and 4-, 2-, and 3-chamber apical views were obtained. The left ventricular diastolic dimension (LVDD) and right ventricular diastolic dimension (RVDD) were measured using Simpson’s biplane formula. We took the measurements for all volumes in triplicate and reported the averages. An independent echocardiography expert reviewed the images and the parameters.

### The study protocol and electrophysiological testing

After 4 weeks, the dogs with MI were randomly categorized into two groups, i.e., MNS group 1 and MNS group 2. The heart was exposed in a pericardial cradle by a median sternotomy performed under anesthesia for the two dog groups. In the MNS group 1, the median nerve in the left forelimbs was exposed, and a pair of bipolar hook electrodes were attached to the nerve. We identified the stimulated nerve as the median nerve by anatomical localization and functional localization of the stimulation effect, and any paw twitches resulting from stimulation were identified as an MNS effect. The electrodes were then connected to a constant current stimulator (S88, Grass Instruments, Quincy, MA, USA) with a stimulus isolation unit (model PSIU6, Grass Instruments) generated 5 Hz, 0.5 ms duration pulses. MNS was performed for 30 min as shown in Figure 2. The lowest voltage level of MNS that attributed to any paw twitches was considered the threshold and then chosen as the voltage for MNS. In the MNS group 2, the bilateral vagotomy was performed before MNS. The methods for MNS were similar to the MNS group 1.

**FIGURE 3:**
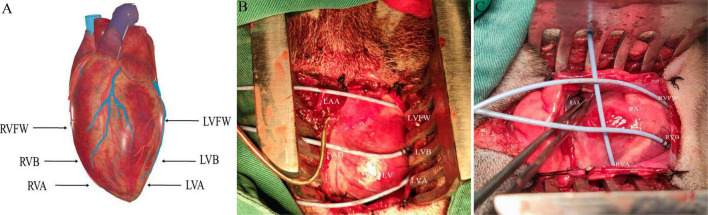
The schematic diagram of electrode placement position during electrophysiological testing **(A)**. **(B,C)** Are electrodes placed in the left ventricle and right ventricle, respectively. LVFW, left ventricular free wall; LVB, left ventricular base; LVA, left ventricular apex; LAA, left atrial appendage; RVFW, right ventricular free wall; RVB, right ventricular base; RVA, right ventricular apex; RAA, right atrial appendage.

The electrophysiological testing was performed before and after MNS. Effective refractory period (ERP) in the right ventricular epicardium at the apex (RVA), free wall (RVFW), and base (RVB) and left ventricular epicardium at the apex (LVA), free wall (LVFW), and base (LVB) were evaluated during ventricular pacing at 300 ms cycle lengths (CLs) using stimuli at twice threshold. Figure 3 shows the schematic diagram of electrode placement position during electrophysiological testing. After every eighth drive stimulus (S_1_), a premature extra stimulus (S_2_) was introduced. As the S_1_–S_2_ intervals approached the ERP, decrements were reduced to 2 ms. ERP was the most prolonged S_1_–S_2_ interval at which S_2_ failed to capture. ERP dispersion was the maximum difference among all sites tested.

The inducibility of VA was conducted before and after MNS and was assessed with programmed ventricular stimulation from the right free wall. Single (S_2_) and double (S_3_) extra stimuli were delivered after eight beats of the ventricular drive (S_1_) at 300 ms (twice threshold, 2 ms duration). To reduce the influence of repeated defibrillation on the results, we measured the ventricular fibrillation threshold (VFT) at the end of the study. After all electrophysiological tests were completed, the VFT was obtained with right ventricular pacing using a train of 30 stimuli (30 ms interval), which was performed at the end of a 20-beat drive train at 300 ms; this ensured that all measurements were taken at the same heart rate. To determine the VFT, we progressively increased the pacing voltage in 1 V steps, with a 30 s rest period before the next pacing train if no VF was induced. The VFT was defined as the minimal voltage required to induce sustained VF.

### Enzyme-linked immunosorbent assay

In this study, 2 ml of venous blood was collected in ethylenediaminetetraacetic acid (EDTA) vacutainers from the MNS group 1 and MNS group 2 dogs before acute MI, before MNS, and after MNS. At the end of the protocol, the animals were euthanized, and the hearts were immediately excised. The control group was assigned to measure the concentration of inflammation cytokines in the tissues. Tissue specimens were obtained from the peri-infarct zone of LV in the MNS group 1, MNS group 2, and the same areas in the control group dogs. The levels of tumor necrosis factor-alpha (TNF-α), interleukin-6 (IL-6), and acetylcholine (Ach) were examined through enzyme-linked immunosorbent assay (ELISA).

### Western blotting

The expression of nuclear transcription factor-κB (NF-κB) and signal transducers in the peri-infarct zone of LV in the MNS group 1, MNS group 2, and the same areas in the control group dogs were measured *via* Western blot (WB). We incubated the membranes with the primary antibody NF-κB (rabbit polyclonal anti-NF-κB antibody, Abcam, E379, Cambridge, UK; used at 1:1,000), then blocked with 5% non-fat dry milk in Tris-buffered saline with Tween 20 (TBST) for 1 h, and incubated with the primary antibody overnight at 4°C. The membranes were then washed in TBST thrice, incubated with the secondary antibody for 1 h at 37°C, and imaged using Immun-Star horseradish peroxidase substrate. The relative expression levels of the proteins were evaluated using image analyzer software (AlphaEase FC, San Leandro, CA, USA).

### Immunohistochemical analysis

Tissues were obtained from the peri-infarct zone of LV for immunohistochemical analysis. We used CD68 (Boster, BA3638, CA, USA) and CD163 (Abcam, ab182422, Cambridge, UK) antibodies for immunohistochemical staining. The tissues were stained in the same session. We determined staining densities by a computer-assisted image analysis system (Image-Pro Plus 3.0, USA).

### Statistical analysis

Data were expressed as the mean ± SD. Two-sample independent Student’s *t*-tests were used to compare means for two groups. ANOVA followed by Newman-Keuls tests were applied to compare the mean values of continuous variables among multiple groups; any significant difference was further analyzed using the Tukey–Kramer test. All of the statistical tests were two-sided, and a probability value of <0.05 depicted statistical significance.

## Results

**FIGURE 4:**
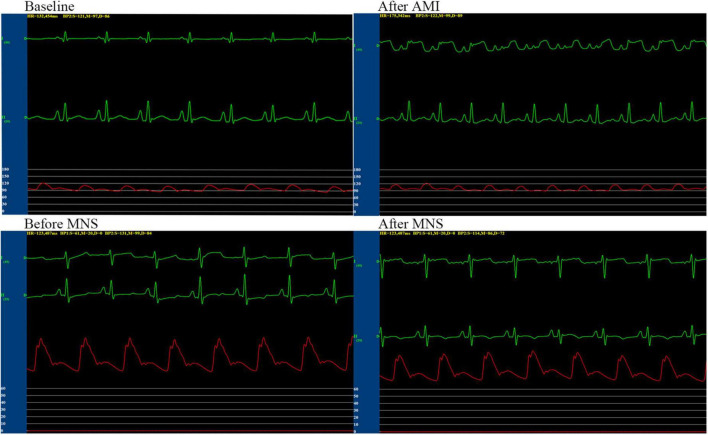
Representative graphs of blood pressure, heart rate, and electrocardiographic monitoring in the MNS1 group dog during baseline, acute myocardial infarction (MI), and before and after 30 min of MSN. MSN, median nerve stimulation.

We performed successful experiments on 13 of 15 dogs with acute MI. Two dogs died during the rearing period. After 4 weeks, six dogs and seven dogs were assigned to MNS group 1 and MNS group 2, respectively. During MNS, the heart rate exhibited a decreasing trend in the MNS group 1 and MNS group 2, but this did not contribute to the significant difference. For instance, the heart rate was 125 ± 12 beats/min before MNS and 121 ± 11 beats/min after 30 min of MNS (*P* > 0.05) in the MNS group 1. The heart rate and blood pressure in the baseline, acute MI, and before and after 30 min of MNS are shown in Table 1 and Figure 4.

**TABLE 1:** Heart rate and blood pressure of experimental dogs in baseline, acute myocardial infarction (MI), and after 30 min of median nerve stimulation (MNS).

	Systolic blood pressure (mmHg) (mean ± SD)	Heart rate (bpm) (mean ± SD)
**MNS group 1**		
Baseline	132 ± 17	136 ± 18
After AMI	152 ± 15	149 ± 16
Before MNS	128 ± 13	125 ± 12
After MNS	122 ± 12	121 ± 11
**MNS group 2**		
Baseline	137 ± 10	129 ± 24
After AMI	154 ± 16	151 ± 21
Before MNS	137 ± 13	128 ± 23
After MNS	136 ± 9	126 ± 21

Blood pressure and heart rate increased after MI compared with baseline, but the difference was not statistically significant. Blood pressure did not change significantly after MNS in MNS1 and MNS2 groups, and heart rate decreased, but the difference was not statistically significant (*P* > 0.05).

### Echocardiography

Compared with baseline, in the MNS group 1 and MNS group 2 dogs, there was a remarkable reduction in the percentage of thickening in the left ventricular anterior walls after 4 weeks; in addition, significantly increased LVDD (the MNS group 1 dogs: 27 ± 2.4 vs. 36 ± 2.9 mm, *P* < 0.01 and the MNS group 2 dogs: 29 ± 2.6 vs. 35 + 2.8 mm, *P* < 0.01) and reduced LVEF (the MNS group 1 dogs: 52 ± 5 vs. 37 ± 6%, *P* < 0.01 and the MNS group 2 dogs: 54 ± 5 vs. 38 ± 5%, *P* < 0.01) were noted. However, there was no significant change in the RVDD between baseline and after 4 weeks in the MNS group 1 and the MNS group 2 dogs. Moreover, we reported no significant differences in the LVDD and RVDD between baseline and after 4 weeks in the control group (Table 2).

**TABLE 2:** Changes in echocardiographic data at baseline and after myocardial infarction (MI) 4 weeks.

	LVDD (mm)	RVDD (mm)	LVEF %
**Control group**			
*Baseline*	28 ± 2.8	12 ± 1.1	55 ± 6
*After 4 weeks*	27 ± 2.6	12 ± 1.2	57 ± 7
**MNS group 1**			
*Baseline*	27 ± 2.4	12 ± 1.3	52 ± 5
*After 4 weeks*	36 ± 2.9^#^	13 ± 1.4	37 ± 6^#^
**MNS group 2**			
*Baseline*	29 ± 2.6	11 ± 1.4	54 ± 5
*After 4 weeks*	35 ± 2.8^#^	12 ± 1.5	38 ± 5^#^

^#^*P* < 0.01 compared with the baseline.

### Electrophysiological testing and ventricular arrhythmia induction

**FIGURE 5:**
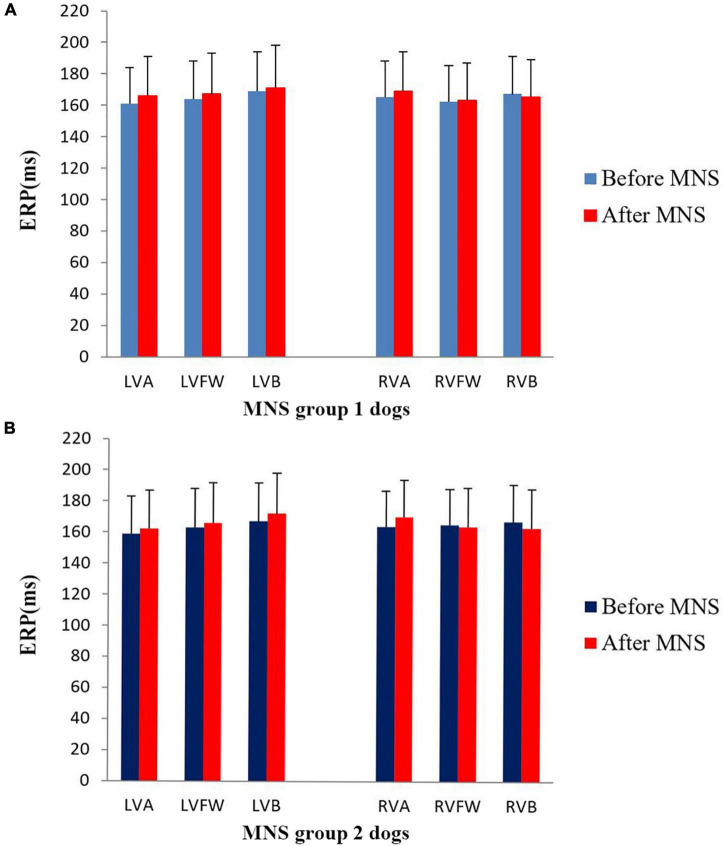
Changes in the ventricular effective refractory period (ERP) before and after median nerve stimulation. **(A)** MNS group 1 and **(B)** MNS group 2. No significant difference in the ERP before and after MNS in the MNS group 1 and MNS group 2 dogs. MSN, median nerve stimulation.

In this study, no significant difference was found in the ventricular ERP between the MNS group 1 and MNS group 2 dogs. Notably, the ERP at RVA was 165 ± 23 ms in the MNS group 1 and 166 ± 19 ms in the MNS group 2, respectively. After 30 min of MNS, the ERPs had no changes at all sites in the MNS group 1 and MNS group 2. For instance, the ERP at the RVA site was 169 ± 25 ms after 30 min of MNS (*P* > 0.05 for all) (Figure 5). Dispersion of ERP had no significant difference between baseline and after 30 min of MNS in the MNS group 1 and MNS group 2 dogs. Programmed ventricular stimulation induced one episode of VT (defined as five consecutive ventricular beats at a rate of 130 bpm) in one dog in MNS groups 1 and 2 and premature ventricular contractions in one dog in MNS group 2 at baseline. After 30 min of MNS, VA was not induced by programmed ventricular stimulation in the two dog groups.

The VFT in the MNS group 1 was significantly higher than that in the MNS group 2 (20.3 ± 3.7 V vs. 8.7 ± 2.9 V, *P* < 0.01). These results demonstrated that it was more difficult to induce VF in the MNS group 1 than in the MNS group 2.

### Enzyme-linked immunosorbent assay

In both MNS groups 1 and 2, the blood concentrations of TNF-α and IL-6 were higher, whereas the level of Ach was lower after 4 weeks compared to the baseline condition. No significant difference was found in the TNF-α, IL-6, and Ach at baseline and after 30 min of MNS in the MNS group 1. However, the levels of the TNF-α and IL-6 were higher, while the level of Ach was lower after 30 min of MNS than baseline in the MNS group 2 (Table 3).

**TABLE 3:** Levels of the tumor necrosis factor-alpha (TNF-α), interleukin-6 (IL-6), and acetylcholine (Ach) in the plasma in control group, median nerve stimulation (MNS) group 1, and MNS group 2.

	TNF-α (ng/ml)	IL-6 (ng/ml)	Ach (ng/ml)
**Control group**			
*Baseline*	0.18 ± 0.05	0.12 ± 0.05	18 ± 4.5
*After 4 weeks*	0.19 ± 0.04	0.15 ± 0.06	20 ± 5.1
**MNS group 1**			
*Baseline*	0.19 ± 0.06	0.11 ± 0.04	18 ± 4.1
*After 4 weeks*	0.31 ± 0.12^#^	0.27 ± 0.09^#^	12 ± 3.1^#^
*After MNS*	0.23 ± 0.11	0.19 ± 0.07	16 ± 4.2
**MNS group 2**			
*Baseline*	0.18 ± 0.07	0.12 ± 0.05	19 ± 4.7
*After 4 weeks*	0.33 ± 0.13^#^	0.29 ± 0.11^#^	12 ± 3.6^#^
*After MNS*	0.33 ± 0.15^#^	0.28 ± 0.10^#^	13 ± 3.7^#^

^#^*P* < 0.05 compared with the baseline.

As highlighted in Table 4, the concentrations of TNF-α and IL-6 in the peri-infarct zone of LV tissues were significantly higher in the MNS group 1 (TNF-α: 0.21 ± 0.06 ng/mg vs. 0.13 ± 0.02 ng/mg, *P* = 0.02; IL-6: 0.16 ± 0.07 ng/mg vs. 0.08 ± 0.02 ng/mg, *P* = 0.03) and the MNS group 2 dogs (TNF-α: 0.30 ± 0.08 ng/mg vs. 0.13 ± 0.02 ng/mg, *P* < 0.01; IL-6: 0.26 ± 0.08 ng/mg vs. 0.08 ± 0.02 ng/mg, *P* < 0.01) than in the control group dogs. However, compared to the MNS group 2, the concentrations of TNF-α (0.21 ± 0.06 ng/mg vs. 0.30 ± 0.08 ng/mg, *P* = 0.04) and IL-6 (0.16 ± 0.07 ng/mg vs. 0.26 ± 0.08 ng/mg, *P* = 0.04) were lower in the MNS group 1 dogs, whereas the levels of Ach were higher in the MNS group 1 dogs than that in the control (18 ± 3.9 ng/mg vs. 12 ± 3.2 ng/mg, *P* = 0.02) and MNS group 2 dogs (18 ± 3.9 ng/mg vs. 10 ± 3.5 ng/mg, *P* < 0.01).

**TABLE 4:** Levels of the tumor necrosis factor-alpha (TNF-α), interleukin-6 (IL-6), and acetylcholine (Ach) in the left ventricular tissues in control group, median nerve stimulation (MNS) group 1, and MNS group 2.

	TNF-α (ng/mg)	IL-6 (ng/mg)	Ach (ng/mg)
Control group	0.13 ± 0.02	0.08 ± 0.02	12 ± 3.2
MNS group 1	0.21 ± 0.06^#^*	0.16 ± 0.07^#^*	18 ± 3.9^#^*
MNS group 2	0.30 ± 0.08^#^	0.26 ± 0.08^#^	10 ± 3.5

^#^*P* < 0.05 compared with the baseline; *P < 0.05 compared with MNS group 2.

### Western blot analysis

**FIGURE 6:**
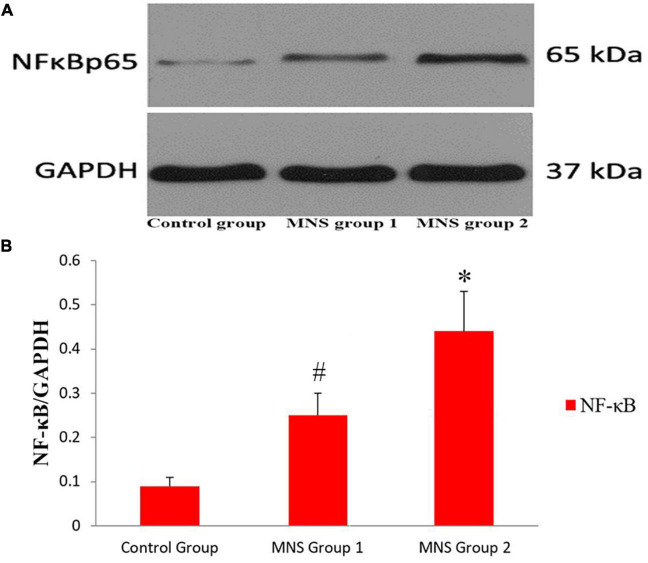
Expression of nuclear transcription factor-κB (NF-κB) protein in the left ventricular tissues. **(A,B)** Representative examples and quantitative analyses of NF-κB expression in the control, median nerve stimulation (MNS) group 1, and MNS group 2 dogs. ^#^*P* < 0.01 vs. control group and MNS group 2; *P < 0.01 vs. control group.

We compared Western blot results of ventricular tissues from the three groups (Figure 6). All immunoblot band intensity measurements were normalized to the intensity of the GADPH band in the loaded sample. Of note, the levels of NF-κB protein in the ventricular samples were significantly higher in the MNS group 2 than in the control group (0.44 ± 0.09 vs. 0.09 ± 0.02, *P* < 0.01) and in the MNS group 1 (0.44 ± 0.09 vs. 0.25 ± 0.05, *P* < 0.01). Compared to the control group, NF-κB protein expression was higher in the MNS group 1 (0.25 ± 0.05 vs. 0.09 ± 0.02, *P* < 0.01).

### Immunohistochemical analysis

**FIGURE 7:**
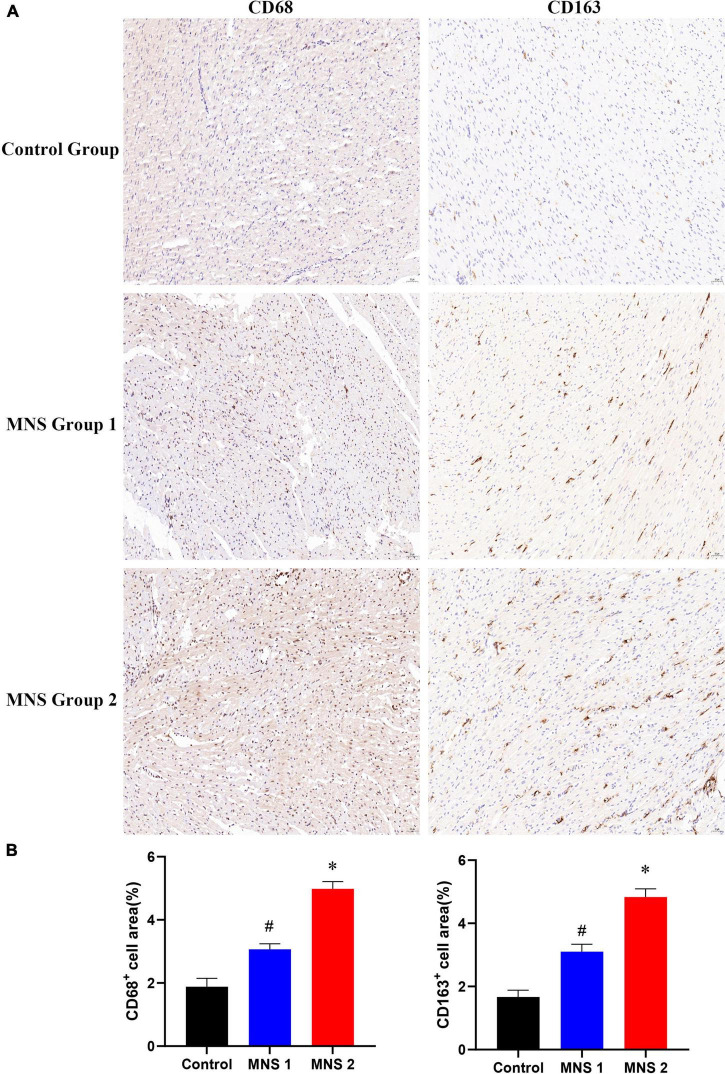
CD68 and CD163 immunohistochemistry (IHC) of left ventricular myocardial tissue in each group. **(A,B)** Representative examples and quantitative analyses of CD68 and CD163 expression in the control, MNS group 1, and MNS group 2 dogs. ^#^*P* < 0.01 vs. control group and MNS group 2; *P < 0.01 vs. control group.

Immunohistochemical staining showed that macrophage markers of CD68 and CD163 were elevated to varying degrees in different groups (Figure 7). The levels of CD68 and CD163 protein in the ventricular samples were significantly higher in the MNS group 2 than in the control group (CD68: 4.98 ± 0.23 vs. 1.88 ± 0.26, *P* < 0.01; CD163: 4.83 ± 0.26 vs. 1.67 ± 0.22, *P* < 0.01) and in the MNS group 1 (CD68: 4.98 ± 0.23 vs. 3.07 ± 0.18, *P* < 0.01; CD163: 4.83 ± 0.26 vs. 3.1 ± 0.24, *P* < 0.01). Compared to the control group, CD68 and CD163 protein expressions were higher in the MNS group 1 (CD68: 3.07 ± 0.18 vs. 1.88 ± 0.26, *P* < 0.01; CD163: 3.1 ± 0.24 vs. 1.67 ± 0.22, *P* < 0.01).

## Discussion

By exploring the influence of MNS on ventricular electrical remodeling and VA vulnerability in a canine model with experimental MI, this study provides evidence for the following: (1) MNS elevates post-infarction VFT and reduces the post-infarction inflammatory response and macrophage infiltration; (2) dissociation of the vagus nerve reverses the above effects of MNS; and (3) cholinergic anti-inflammatory pathways may play an important role in the effects of MNS on VFT.

Several compelling bodies of evidence have shown that the risk of SCD after MI is highest during the first 30 days (1, 16, 17). The sympathetic nerve sprouting at the peri-infarct zone after MI perhaps plays a vital role in the incidence of SCD. Recently, decreased sympathetic nerve activity, such as renal denervation or low-level vagus nerve stimulation, was found to potentially assist in VA suppression (18, 19). In other previous studies, Li et al. (14) and Zhu et al. (20) demonstrated that MNS inhibits cardiac sympathetic activity during myocardial ischemia. Elsewhere, Lujan et al. found that MNS decreases ischemia-reperfusion-induced VT by lowering cardiac metabolic demand (15). However, the effect of MNS on VA vulnerability after long-term MI remains elusive.

In this study, we observed the effects of MNS on VA vulnerability in a canine model with MI for 4 weeks. Of note, it was revealed that the ventricular ERP and VT vulnerability had not changed obviously. However, the VFT increased significantly after 30 min of MNS. Furthermore, TNF-α, IL-6, and NF-κB levels in the peri-infarct zone of LV decreased after MNS. Interestingly, the effects of MNS on the VFT and ventricular inflammation factors were suppressed by bilateral vagotomy. In our previous study, we found that MNS not only attenuated left stellate ganglion nerve activity but also enhanced cardiac vagal nerve activity (13). Collectively, these results further demonstrated that the increased VFT and decreased ventricular inflammation factors by MNS are closely associated with cardiac vagal nerve activity. The anatomical pathways pertaining to these effects are most likely associated with the vagal networks, in particular, vagal brainstem nuclei such as the nucleus tractus solitarius (NTS) and dorsal motor nucleus of the vagus (DMV) (21).

Previous studies demonstrated that the levels of inflammation factors increased after MI, and vagal nerve stimulation could inhibit the inflammatory reaction and relieve myocardial ischemia/reperfusion injury by activating the cholinergic anti-inflammatory pathway (22, 23). The anti-inflammatory activity of the vagal nerve activity is linked to NF-κB signaling (24). NF-κB is presently considered a key transcription factor, regulating the expression of pro-inflammatory cytokines in signal transduction pathways. Based on the present findings, we suggest that the inhibition of NF-κB activation is associated with a better outcome post-MNS.

In clinical practice, the stimulation of the Neiguan spot has been utilized to treat MI (25). The Neiguan spot is located in the portion of the meridian of the heart minister situated in the forearm, along the course between the two tendons. This acupoint overlies the trunk of the median nerve. Therefore, the clinical application of acupuncture at the Neiguan point, which is close to the median nerve, in patients with MI also indirectly confirms the findings of our study. Above all, we speculated that MNS could suppress SCD in patients with MI by activating the cholinergic anti-inflammatory pathway.

## Study limitations

This study has some limitations. First, we only explored the effect of 30 min of MNS on ventricular electrophysiology and VT vulnerability in a canine model with MI. No significant changes were found in ventricular ERP. Therefore, the effects of long-term intermittent or stronger MNS on ventricular electrophysiology and VT vulnerability are unclear. Second, previous studies revealed that local heterogeneity of refractoriness is more significant in the peri-infarct zone than remote zone, and widely different monophasic action potential among the epicardial, mid-myocardial, and endocardial layers increased transmural heterogeneity of repolarization after MI. Moreover, we did not investigate the changes in transmural action potential during MNS. Whether MNS influences the transmural action potential after MI is unknown. Third, our previous study demonstrated that MNS attenuated stellate ganglion nerve activity and enhanced vagal nerve activity, whether MNS affects the tissue of vagus nerve and ganglion plexus-related changes is unknown, and harvesting cardiac ganglion plexus tissue for relevant assays will help further analysis. Fourth, monitoring imaging for the cardiac mapping and the hypo voltage area can better understand the mechanism of MNS in cardiac electrophysiology.

## Conclusion

This study revealed that MNS increases VFT induced by a train of rapid stimulation post-MI. Additionally, bilateral vagotomy suppresses the effects of MNS on VFT; these findings validate the hypothesis on whether the effects of MNS on VFT may be associated with the cholinergic anti-inflammatory pathway.

## Data availability statement

The original contributions presented in the study are included in the article/supplementary material, further inquiries can be directed to the corresponding author.

## Ethics statement

The animal study was reviewed and approved by Institutional Animal Care and Use Committee of the Renmin Hospital of Wuhan University. Written informed consent was obtained from the owners for the participation of their animals in this study.

## Author contributions

QZ performed the conception or design of the work. XW, YQ, YY, YW, YZ, and SZ contributed to the acquisition, analysis, and interpretation of data for the work. XW and YQ drafted the manuscript. QZ and YY critically revised the manuscript. All authors gave final approval and agreed to be accountable for all aspects of the work ensuring integrity and accuracy.
